# Chemists and Their Work

**Published:** 1888-12-15

**Authors:** 


### Chemists and their Work.

A former paper discussing the mutual relations of doctors and chemists advanced the principle that the sole province of the latter lay in the vending and dispensing of drugs. The ground was strongly taken up that the chemist who ventured to prescribe was likely to do injury to the public, besides committing an illegal act and trespassing on the corporate rights of the medical profession. The present article is offered to readers lest a wrong impression should have been conveyed as to the difficulties that surround the art of modern dispensing, or lest it should appear that the statements of the writer were based on imperfect knowledge.

To the question, "What are the qualities necessary to a good dispenser?" one would answer somewhat as follows: "The perfect dispenser must be careful of his dress, appearance, and manners; his habits must be those of order and cleanliness; he must be conscientious as to the quality of his drugs, so that they be of the best; he must have a thorough knowledge of *materia medica, i.e.*, of the properties, preparations, and uses of all substances employed in medicine; he must be skilled in chemical actions and combinations of drugs, so as to avoid incompatibles and inert or dangerous compounds; he should be practised in the interpreting of foreign and of ill-written prescriptions; finally, he must keep abreast of new discoveries, and should bring to bear on all things connected with his craft a trained judgment, a high special knowledge, and a most scrupulous care."

That seems a long list of virtues, but it is none the less a fact that anyone who aspires to be a master in the art of dispensing must possess all, or nearly all, those qualities, the truth of which assertion will be shown by further inquiry. Turning to the first point of neatness of dress, one may safely say there is hardly any trade which demands more attention to externals than that of the chemist. The handling of powerful drugs is a serious matter, in which carelessness may at any time endanger life, and everyone knows how people are influenced by outside appearances. Hence the care which the first-rate chemist bestows on all details connected with his shop, such as the dress and manners of his assistants and the neatness with which he sends out his wares to customers. An authority says on this point: "The dispenser who economises on his drugs is a rogue, but he who economises on his bottles, corks, pill-boxes, or paper is a fool. . . . Patients are apt to form an estimate of the qualities of the compounder of a prescription from the style of his penmanship, reasoning that if he is careful, clean, and neat in one particular, of which they are competent to judge—*i.e.*, the handwriting on the label—the compounder must exercise similar qualifications in the dispensing, upon which they cannot hope to pass judgment."

The same unwearying attention to details is called for throughout the whole practical part of the dispenser's business, involving, as it does, the handling of so many different kinds of substances. There are processes of preparation without end, maceration, percolation, infusion, decoction, filtering, pounding in mortars, measuring, weighing, evaporating, precipitating, and a host of other mysteries. Indeed, it would no little astonish one of the ancient alchemists—the fathers of modern chemical science—could he but view for a short space the every-day work of a nineteenth-century druggist's shop. The ground covered by the pharmacist is extensive, for he has to deal with minerals, gums, seeds, roots, fruits, oils, extracts, fats, acids, alkalies, powders, poisons, salts, alkaloids, aromatics, spices, explosives —in short, with material of nearly every conceivable kind. It goes without saying that he must have an exact knowledge of weights and measures, not only of his own, but also of foreign countries. The interesting fact may be mentioned here that *minims* are usually measured out where *guttæ* or "drops" are prescribed, the reason for this being that the size of a drop may vary greatly with the nature of the liquid, the temperature, the lip of the bottle, and other conditions. Thus, it is stated that chloroform dropped from an ordinary phial will at one time give 150 and at another 300 drops for a fluid drachm.

But above and beyond the purely technical details of his trade, the dispenser will be constantly called upon to exercise both common-sense and judgment. For instance, he has often to decide what particular preparation of a drug is meant from the date of the prescription, the reason being that from time to time there appears a fresh edition of "The British Pharmacopœia." That book is the authority on the subject, and as there are changes in every issue, old preparations being altered or cut out, and new ones added, the dispenser has often to be guided entirely by the year in which the prescription was written. Then the physician may shorten the name of a drug in such a way that it will bear two or three interpretations. Thus "*aq: menth:*" may mean *aquae menthae piperitae*, peppermint water, a flavouring agent in daily use, or, *aquae menthae viridis*, spearmint water, which is seldom ordered. In this case the chemist would probably decide on the more commonly used of the two preparations, but as both are harmless, little mischief would ensue even if the prescriber's exact intention were not carried out. At other times, however, these abbreviations—quite unnecessary, be it remarked—may leave the door open for serious mistakes. Thus "*hyd: chlor:*" may mean either calomel, or corrosive sublimate, or chloral, and it must be left to the common sense of the dispenser to determine which is really meant. As a last resort, it is always open for him to consult the writer of the prescription, if at hand, but there are obvious objections against such a course. A pharmacist remarks, "There are cases where the dispenser must use his own discretion and be guided by experience in trying to find out the intentions of the prescriber, and to do this he must go beyond the bare written instructions. If he does this with tact and to the best of his ability, he will ensure the confidence of the practitioner and his patient, and avoid giving any explanation."

The common occurrence of prescriptions that need alteration at the hands of the chemist points to a defect in medical education, for there can be little doubt that the student leaves the schools, as a rule, quite unpractised in this branch of his profession. The modern tendency is happily towards practical teaching, instead of the cramming systems now in vogue, and some forcible observations on this point were lately made by Sir Andrew Clarke at Sheffield. "This is the age of examinations, and for qualifications in the various departments of science and art the questions to be answered are so numerous, recondite, and complex, that the kind and degree of preparation necessary to answer them are becoming incompatible with true education, genuine study, and thorough work. Much, then, we must sympathise with the teacher who is forced to follow a fashion which his experience condemns, and to leave comparatively unused those higher methods of teaching which he knows and trusts."

A great and, as it would seem in many cases, an unfair responsibility is thrown upon chemists in determining whether unusual or dangerous doses should be dispensed. By English law the chemist is responsible for accidents that may occur from the making up of such prescriptions, however illegible they may be. In the German pharmacopœia a table of maximum doses of certain powerful medicines is given, and if the prescriber desire to exceed the quantities there set down he is required to mark the quantity. If this be not done, the dispenser is held responsible for the consequences if he dispense the dangerous dose. "Two curious cases are mentioned by Dr. Hager. In one an extra dose of cyanide of potassium had been ordered, and the prescriber had several times *underlined* the quantity. The patient died, and the dispenser was condemned to a year's imprisonment because he had dispensed the medicine without the proper mark (!) being attached. In the other case, the physician meant to order 4 grammes of chloral hydrate, and he should have written grm. 4·0. He omitted the decimal point, however, and the dispenser gave 40 grammes. At that time chloral hydrate was not included in the German pharmacopœia, and therefore was not in the table of dangerous substances. But the dispenser was sentenced to a long term of imprisonment."

As to the minute special knowledge required in the various methods of dispensing, it is impossible to do more than hint at it here. Suffice it to say that every step in the making of pills, tablets, capsules, powders, ointments, plaisters, mixtures, emulsions, and other preparations, fairly bristles with details, all of which must be observed by the good workman. The reader will find further information in a little volume published by the *Chemist and Druggist*, entitled "The Art of Dispensing," from which, indeed, the materials of this sketch have been largely drawn.

The handwriting of doctors is notoriously bad, and a passage on the subject of illegible prescriptions is so good that one may be excused in quoting it at length: "The capability of pharmacists to decipher illegible caligraphy is so generally known as to be almost proverbial. It is a kind of expertness which they have acquired through long practice in reading the prescriptions of physicians. Their business requires this art; it has received official recognition by being made a part of the requirements of the qualifying examination, at which badly-written medical prescriptions have to be read by candidates, and teachers find it necessary to collect specimens of bad medical penmanship on behalf of their pupils.

"The duty of the dispenser who has an illegible prescription presented to him has never been clearly defined; he has certainly a perfect right, legally, to refuse to compound a prescription which he cannot read; but it is believed that in the case of prescriptions which have previously been dispensed he is justified in doing his best. The best, however, may be a serious matter to the patient if it happens to be contrary to the intentions of the prescriber. It is far better for the dispenser that he should not risk his own reputation or the comfort of his customer by undertaking a task respecting which he is uncertain."

In concluding, one may hope that the evidence laid before the reader will have gone far to establish the statement made at the outset as to the qualities requisite to a good dispenser. At any rate, it will be clear there is plenty in the legitimate work of the chemist to require all his time and energy. While fully recognising the constant claims on the technical skill, the special knowledge, the judgment, and the responsibility of the dispenser, yet one adheres firmly to the previously-expressed opinion as to the inadvisability of his trespassing into the province of the qualified medical practitioner, by prescribing as well as vending drugs.

